# Role and outcomes of community health workers in HIV care in sub-Saharan Africa: a systematic review

**DOI:** 10.7448/IAS.16.1.18586

**Published:** 2013-09-10

**Authors:** Grace W Mwai, Gitau Mburu, Kwasi Torpey, Peter Frost, Nathan Ford, Janet Seeley

**Affiliations:** 1 Division of Medical Education, Brighton and Sussex Medical School, University of Brighton, UK; 2 International HIV AIDS Alliance, Preece House, Hove, East Sussex, UK; 3 Division of Health Research, Lancaster University, Lancaster, UK; 4 Family Health International, Garki, Abuja, Nigeria; 5 Centre for Infectious Disease Epidemiology and Research, University of Cape Town, South Africa; 6 School of International Development, University of East Anglia, Norwich, UK; 7 MRC/UVRI Uganda Research Unit on AIDS, Entebbe, Uganda

**Keywords:** HIV, care, community health workers, sub-Saharan Africa, systematic review

## Abstract

**Introduction:**

The provision of HIV treatment and care in sub-Saharan Africa faces multiple challenges, including weak health systems and attrition of trained health workers. One potential response to overcome these challenges has been to engage community health workers (CHWs).

**Methodology:**

A systematic literature search for quantitative and qualitative studies describing the role and outcomes of CHWs in HIV care between inception and December 2012 in sub-Saharan Africa was performed in the following databases: PubMed, PsychINFO, Embase, Web of Science, JSTOR, WHOLIS, Google Scholar and SAGE journals online. Bibliographies of included articles were also searched. A narrative synthesis approach was used to analyze common emerging themes on the role and outcomes of CHWs in HIV care in sub-Saharan Africa.

**Results:**

In total, 21 studies met the inclusion criteria, documenting a range of tasks performed by CHWs. These included patient support (counselling, home-based care, education, adherence support and livelihood support) and health service support (screening, referral and health service organization and surveillance). CHWs were reported to enhance the reach, uptake and quality of HIV services, as well as the dignity, quality of life and retention in care of people living with HIV. The presence of CHWs in clinics was reported to reduce waiting times, streamline patient flow and reduce the workload of health workers. Clinical outcomes appeared not to be compromised, with no differences in virologic failure and mortality comparing patients under community-based and those under facility-based care. Despite these benefits, CHWs faced challenges related to lack of recognition, remuneration and involvement in decision making.

**Conclusions:**

CHWs can clearly contribute to HIV services delivery and strengthen human resource capacity in sub-Saharan Africa. For their contribution to be sustained, CHWs need to be recognized, remunerated and integrated in wider health systems. Further research focusing on comparative costs of CHW interventions and successful models for mainstreaming CHWs into wider health systems is needed.

## Introduction

An estimated 33.3 million people are living with HIV globally, 67% of whom reside in sub-Saharan Africa. In addition, the region accounted for almost 70% of 1.7 million HIV-related deaths that occurred in 2011 [1]. Although the impact of HIV in this region varies widely [2], there is evidence that scale-up of antiretroviral therapy (ART) can reverse some of the negative social economic impacts of HIV on individuals, families and communities, with positive changes in life expectancy [3], demographic composition [4] and fertility [5].

Efforts to scale up HIV treatment and care in sub-Saharan Africa over the past decade, while successful, have exposed pre-existing weaknesses of health systems in this region [6], in particular the lack of health workers to provide ART [7, 8]. Sub-Saharan Africa has just 3% of the global health workforce [9], and faced with a shortage of skilled health workers attention has turned to the potential for lay health workers, such as peer educators, home-based carers, lay counsellors, adherence counsellors, health extension workers, community caregivers and others, to provide HIV care [10–12].

Community health workers (CHWs) have already been widely adopted in many settings, either to complement the work of formally qualified health workers or to assume specific responsibilities. The approach has generated debate regarding the types, roles and management of CHWs. Questions have arisen regarding whether they should work in communities or within facilities, who should select them and to whom they are accountable [13, 14]. Although these issues remain important, a recent review suggests that some of the universal features of CHWs include their comparatively limited training, diverse typologies and titles that may or may not be directly related to their roles and their primary focus on communities [15].

CHWs can play important roles in responding to other diseases. In tuberculosis (TB), they support treatment adherence, and screening and education activities, often with positive impact on treatment success [11, 16]. CHWs can be deployed to assess, classify and treat malaria and pneumonia among children, and to promote immunization and breast feeding, and can have positive effects on child, neonatal and adult morbidity and mortality [11, 17–20]. In maternal and child services, CHWs provide ante- and postnatal care, advise on family planning and childhood nutrition, increase facility-based deliveries, conduct home visits, formulate birth plans, facilitate home deliveries and respond to obstetric emergencies [21–24]. There is some evidence that CHWs could be a less costly approach for the delivery of certain services such as malaria case management among others [25, 26]. Considering this positive track record in areas of health care delivery, and the insufficiency of trained health professionals to deliver ART, in 2008, the World Health Organization endorsed task shifting to allow lower cadres of health workers assume greater responsibility in HIV care delivery [27].

Despite their proliferation in HIV programs, the outcomes of CHWs are neither widely understood nor consistently documented, leading to a lack of harmonized approach to their utilization, remuneration and retention. There are divergent views about the value and position of CHWs in relation to formal health systems [13, 15]. Although some view them as an essential component of the health care system [10, 12, 13, 27–29], others are more critical of their role [30]. In particular, a considerable body of literature addresses specific concerns related to task shifting to CHWs – as distinct from task shifting to nurses and non-physician clinicians. This literature embodies recent discourses regarding CHWs' contribution to patient outcomes and quality of care [31], as well as more general issues related to their appropriateness in different contexts [32], their remuneration and sustainability [33], cost-effectiveness [34], competencies, training and quality assurance [35]. Although some have argued that task shifting is an efficient mechanism of mobilizing the unexploited community resource to provide health care [9], others warn that there is a risk of CHWs being seen as “just another pair of hands” [36], with no linkage to formal health systems [15]. These concerns may partly explain the reluctance by some national ART programs to embrace task shifting of HIV care to CHWs [37]. Lacking formal recognition, CHWs are often inadequately resourced and undervalued [38].

Two recent systematic reviews of task shifting [12, 31], conclude that task shifting could reduce cost and improve access to services without compromising patient outcomes. These reviews examined the effectiveness of shifting tasks from doctors to other health providers including nurses, non-physician clinicians and community workers, and did not specifically focus on the role and outcomes of CHWs. This distinction is important because the range of skills and training differs significantly between CHWs on the one hand and nurses and clinicians on the other, which could translate to differences in outcomes. This systematic review focuses specifically on roles and outcomes of CHWs in HIV programs in sub-Saharan Africa.

## Methods

This review considered evidence from both qualitative and quantitative studies [39], followed the reporting requirements of the PRISMA guidelines [40] and used an aggregative narrative synthesis approach to summarize the results [41].

### Definitions

For the purposes of this review, a CHW was defined as “any health worker who performs functions related to health care delivery; has trained in some way in the context of the intervention; and has no formal professional or paraprofessional certificate or degree in tertiary education” [11]. The varying titles of CHWs used in the reviewed studies are included in Table 1.

**Table 1:** Summary of included studies

#*	Paper	Setting	Intervention*	Methodology	Sample**	% Female	Duration	Roles of CHWs***	Outcomes
1	Alamo *et al*., 2012	Uganda	CATTS	Mixed [retrospective data review (patients) and qualitative interviews (CATTS)]	347 patients; 47 CATTS	71; 63	26 months	Home visits; adherence counselling; referrals to PMTCT; feedback to clinicians on patients’ health status	Timely patient referrals that reduced delays in care
2	Apondi *et al*., 2007	Uganda	Field officers	Cohort study	654 patients	72	12 months	Home visits; drug delivery; patient monitoring	Reduced stigma and improved family support and relationships
3	Arem *et al*., 2011	Uganda	Peer health workers	Mixed (qualitative interviews, direct observation and focus group discussions; quantitative surveys, data analyses on virologic outcome)	36 peer health workers; 12 patients; 10 clinic staff	NR; 50; NR	24 months	HIV counselling; adherence support through home visits, pill counting and patient tracing; registering patients	Reduced stigma; improved retention; reduced workload, so more time for clinic staff to attend to other patients; improved medical care access, clinic organization, patient flow, patient–provider communication
4	Chang *et al*., 2010	Uganda	Peer health workers	RCT	1336 patients	67	26 months	HIV counselling; adherence support through home visits and pill counting	Reduced virologic failure rates at 96 weeks and reduced loss to follow-up
5	Gusdal *et al*., 2013	Ethiopia and Uganda	Peer counsellors	Qualitative	79 patients; 17 peer counsellors and 26 health care workers	58; NR; NR	3 months	Adherence support by acting as role models; assisting disclosure of HIV status; linking clients and clinics; home visits (personal hygiene and household chores); enrolment in food support programmes	Improved confidence; dispelled myths about HIV and ART
6	Grimwood *et al*., 2012	South Africa	Patient advocates	Cohort study	3563 children	49	36 months	Adherence and psychosocial support for children's caregivers; home visits; HIV education and health promotion	Improved patient retention and survival after three years of ART: 91.5% (95% CI: 86.8–94.7%) vs. 85.6% (95% CI: 83.3–87.6%) among children without patient advocates
	Igumbor *et al*., 2011	South Africa	Patient advocates	Retrospective cohort study	540 patients’ records	64	40 months	ART adherence support; adherence counselling; assisting disclosure of HIV status	Better viral suppression at six months of treatment; better retention in care; higher proportion of patients with patient advocates (89%) attained a treatment pickup rate of over 95% (67%; *p*=0.021); better disclosure rates among patients with patient advocates (58%) than those without (42%; *p*=0.005)
7	Jaffar *et al*., 2009	Uganda	Field officers****	RCT	1453 patients	71	42 months	Screening for drug toxicity and disease progression; adherence support during home visits	Similar mortality and viral suppression outcomes between home-based and faculty-based ART provision. However, the later was cheaper: USD 793 versus USD 838
8	Johnson and Khanna, 2004	Kenya	CHWs	Qualitative interviews, focus group discussions and participant observation	46 key- informantsand 9 youths	NR	3 months	Personal care (meal preparation, household chores, giving medication, needs assessment for supplies); income generation; behaviour change communication on wife inheritance; HIV education and counselling	Improved quality of life, dignity and sense of belonging; positive perception of people living with HIV (reduced stigma)
9	Joseph *et al*., 2012	Lesotho	Lay health workers	Qualitative (ethnographic interviews and participant observation)	30 lay health workers	43	30 months	Translation; home visits; pre-test and adherence counselling; patient triage; medication and food distribution; laboratory specimen processing; assessment of vital signs; maintaining registers; decision-making regarding organisation of clinics	Improved patient flow at clinic; locals more comfortable with foreign doctors
10	Kipp *et al*., 2011	Uganda	Lay community volunteers	Cohort study (quasi-experimental)	385 patients	58	48 months	Home visits; monitoring ART adherence through pill counts; assessment for drug reactions; referral and supplying patients with ARVs	Better ART adherence, improved quality of life and viral suppression at community level; all seven children born were HIV negative. However, loss to follow up was higher in the intervention group compared to the control group (24.9% vs. 15.5%).
	Kipp *et al*., 2012	Uganda	Community volunteers	Cohort study (quasi-experimental)	385 Patients	58	60 months	Home visits; supplying ARVs; monitoring adherence through pill counts; condom distribution; education on HIV prevention; assessment for opportunistic infections and drug reactions; referral; data collection and recording on standardized forms	Increased access to ARVs at community level; 93% virologic suppression at community level versus 87.3%; *p*=0.12.
11	Rich *et al*., 2012	Rwanda	CHWs	Cohort study	1041 patients	67	24 months	Directly observed therapy; TB and nutrition screening; education on side effects and opportunistic infections; social support and companionship	92.3% of patients were retained in care, 5% died and 2.7% were lost to follow-up; better virologic outcomes at 24 months
12	Sanjana *et al*., 2009	Zambia	Lay counsellors and health workers	Mixed (interviews, focus group discussions, review of two-year records pre and post deployment of CHWs)	19 lay counsellors; 121 health workers; 1083 register entries	42; NR	24 months	Counselling	Less waiting time for counselling; 70% of counselling offered by CHWs; lower error rate in medical records filled by lay counsellors (6.44/1,000 fields) than health care workers (16.81/1,000 fields)
13	Schneider *et al*., 2008	South Africa	CHWs	Qualitative	260 CHWs	92	21 months	Counselling; follow up; HIV education	Increased ART uptake
14	Selke *et al*., 2010	Kenya	Community care coordinators	Cluster RCT	239 patients	73	12 months	Data collection using personal digital assistant on symptoms and vital signs; monitoring ART adherence; assessing for opportunistic infections, food security and domestic violence; dispensing ARVs	ART adherence in the intervention group was 95% vs. historical 80% in the control group; patients in the intervention arm made fewer clinic visits *p*<0.001
15	Simon *et al*., 2009	Mozambique	CHWs	Qualitative (participant observation)	138 CHWs	NR	13 months	Directly observed therapy support; defaulter tracing; HIV education; collecting ARVs; referral for TB/HIV, diarrhoea and malaria; income generation (gardening)	Increased reach of services to the community; timely referrals
16	Suri *et al*., 2007	South Africa	CHWs	Mixed (cross-sectional survey, interviews, focus group discussions)	120 CHWs	NR	<1 month	Directly observed therapy; TB and HIV surveillance; education; health promotion; data collection	No outcomes explored
17	Torpey *et al*., 2008	Zambia	Adherence support workers	Mixed (cohort, retrospective record review, focus group discussions, interviews)	500 patients; 8875 clinic records	59	24 months	ART adherence counselling; education on advantages of ART	Loss to follow-up rates declined from 15% to 0%; adherence support increased from 0% to 55.2%; reduced clinic waiting times and workload for health care workers
18	Uys, 2002	South Africa	CCGs	Mixed (cross-sectional survey, interviews, participant observation)	16 CCGs	99	18 months	Home-based hygiene and wound care; health education; HIV, bereavement, partner and succession counselling; directly observed therapy support	Enhanced the dignity of and quality of care for people living with HIV
19	Wouters *et al*., 2009	South Africa	CHWs (people living with HIV on ART)	Cohort study	268 patients	67	36 months	ART adherence and counselling; lifestyle and disclosure counselling	Increased motivation to adhere to ART; improved quality of care; CHW support improved disclosure to family members
20	Zachariah *et al*., 2006	Malawi	Home-based care volunteers, lay counsellors, family caregivers	Cohort study	5106 patients, 1362 family caregivers	NR	24 months	HIV counselling and testing; adherence counselling; referral for ART; TB screening; income generation; PMTCT referrals for HIV-positive mothers	Lay counsellors conducted 41% of all HIV testing in the district; coverage of home-based care services increased by 39%; earlier diagnosis and treatment of TB
21	Zachariah *et al*., 2007	Malawi	Community volunteers and patients	Cohort study	1634 patients	36.5	18 months	Supplying cotrimoxazole for opportunistic infections; family caregiver support; referral of those with drug reactions; adherence counselling; tracing defaulters; nutritional support; social mobilization; orphan support; income generation	Community support reduced risk of death RR [0.22 (0.15–0.33)], loss to follow-up OR [0.02 (0–0.12)] and stopping ART [0.23 (0.08–0.54)]; one patient lost to follow-up in areas with community support compared to 39 in areas without: *p*<0.001

* Four cohort publications related to two patient cohorts and were therefore treated as the same studies.

** Sample size in qualitative studies refers to CHWs interviewed; in cohort studies and RCTs it refers to HIV patients; in mixed and cohort studies it refers to key informants such as clinic managers as well as patients and CHWs.

*** The roles of other health professionals were reported in some of the studies, but were not the focus of this review.

**** In this study some field officers had degree qualifications or college diplomas.

Abbreviations: ART=antiretroviral therapy; ARVs=antiretroviral drugs; CATTS=community ART and TB treatment supporters; CCGs=community caregivers; CHWs=community health workers; PMTCT=prevention of mother to child transmission of HIV; NR=not reported; RCT=randomized clinical trial; TB=tuberculosis.

### Search strategy and study selection

The following databases were searched from inception to December 2012 for articles published in English or French: PubMed, PsychINFO, Embase, Web of Science, JSTOR, WHOLIS, Google Scholar and SAGE Journals online [39]. We used a compound search strategy combining truncated and non-truncated terms to identify CHWs, such as “health aides”, “community health workers”, “lay health workers”, “health volunteers”, “health communicators”, “adherence supporters” and “health guides”. We combined these with appropriate terms identifying the role, impact or effectiveness of CHWs, such as “role”, “professional”, “family health” and “social change” using the Boolean operator: “AND” or “OR” as appropriate. These were also applied as MeSH terms in PubMed (Supplementary file 1). We also included a variety of research types, using the terms “evaluation studies”, “comparative effectiveness research”, “programme evaluation”, “cost–benefit analysis”, “effectiveness” and “impact” using the Boolean operator: “OR”, as appropriate, in order to identify empirical studies exploring CHWs outcomes (Supplementary file 1). Truncation was applied mainly in Web of Science and Embase. “Related citations” and “cited by” links in PubMed and Google Scholar were used to identify potentially relevant studies. Bibliographies of all included and closely related but excluded studies were also searched for additional relevant citations.

### Inclusion and exclusion criteria

Titles and abstracts of all references were screened by the first author using pre-defined criteria; that is, studies were included, if they clearly stated the research methodology used, documented CHWs role or outcomes in the HIV response in sub-Saharan Africa, and were published in English or French. A random selection of 25% of titles was independently screened by the second author. No studies were excluded based on quality, but limitation of evidence from such studies was highlighted in the results and discussion.

### Quality assessment

Quality assessment extracted data on sampling approach, study design, use of objective outcomes, sources of bias and generalizability of findings using separate criteria for qualitative studies [42], randomized controlled trials (RCTs) [43] and observational studies [44]. Using these criteria, studies were classified as good quality, moderate quality, or poor quality (Supplementary file 2). The first author applied the checklist to all studies, whereas the second author independently reviewed 25% of randomly selected studies using the same checklist for quality control purposes.

### Data extraction and analysis

Data were extracted using a predetermined extraction form. Analysis of outcomes of interest (roles and outcomes of CHWs) was then performed by identifying emerging themes from the extracted data and categorizing them using narrative synthesis approach [41, 45]. Quantitative studies were also analyzed by a narrative commentary on the observed associations, with reference to *p-*values and confidence intervals of observed associations, and in relation to their strengths and limitations. Due to the heterogeneity of data, meta-analysis was not performed [39]. To determine how the roles and outcomes of CHWs from different papers were related to one another, we mapped out the roles of CHWs each step along the “continuum of care”, which describes the cascade of steps through which an individual gets diagnosed with HIV, is linked to relevant services, remains adherent to ART and is retained in HIV care for better outcomes in relation to viral load, morbidity, mortality and quality of life [46].

## Results

### Characteristics of included studies

**Figure 1:**
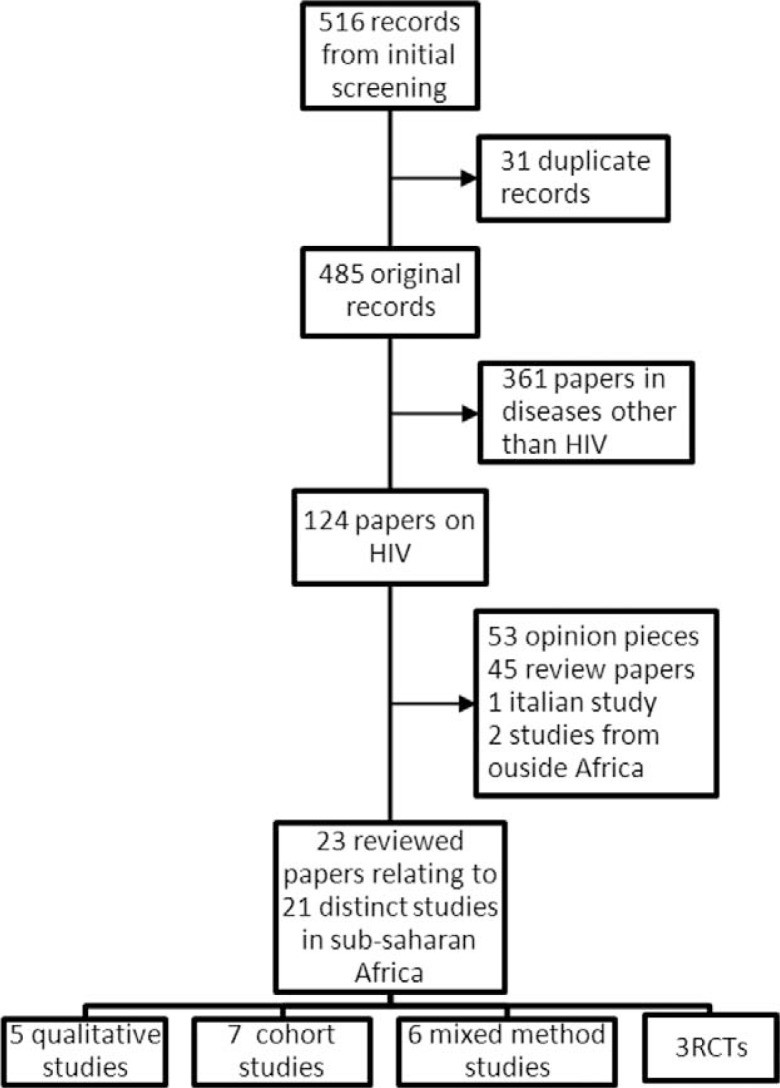
Schematic representation of study selection.

The electronic search identified 516 records, from which 23 papers describing 21 studies were retained (Figure 1). Publications relating to the same cohort were treated as one study even though they were separate manuscripts. Overall, the search strategy criteria were quite specific. As a result, the review had a total of 21 distinct studies. These comprised five qualitative studies, seven cohort studies (nine papers), three RCTs and six mixed-method studies in nine countries in east and southern Africa: Ethiopia, Kenya, Lesotho, Malawi, Mozambique, Rwanda, South Africa, Uganda and Zambia.

### Quality of included studies

Overall, studies were rated as being of moderate quality. The majority were non-randomized and non-comparative. The main limitation of the three RCTs related to their lack of complete blinding, which is difficult to achieve in trials for community-based interventions [47]. In these instances, where quality criteria were “not applicable” to some included studies, such as blinding allocation to CHWs interventions, these criteria were disregarded. Limitations of the cohort and qualitative studies related to high rates of loss-to follow up, lack of important baseline data, subjective outcome measures and limited generalizability (Supplementary file 2).

### Roles and outcomes of community health workers in sub-Saharan Africa: emerging themes

A narrative synthesis of the findings identified two main themes related to the effect of CHWs on 1) patient outcomes and 2) health systems, based on the specific activities that CHWs engage in. These effects of CHWs on health systems and patient-level outcomes were not mutually exclusive, but were often inextricably linked (Table 2).

**Table 2:** Roles and outcomes of community health workers in HIV care in sub-Saharan Africa

Themes	Sub-themes	Activities	Examples of outcomes
Patient-oriented themes	Knowledge, education and literacy	HIV and stigma education Drug readiness training Infection control education	Positive perception of people living with HIV
	Behaviour change and other counselling	HIV pre-test counselling Partner/couple counselling Behaviour change counselling Disclosure counselling Bereavement counselling	Increased uptake of HIV testing; Improved disclosure; Improved motivation to be tested for HIV
	Referral	Referral for co-infections, such as TB Referral for ART, voluntary counselling and testing, PMTCT, opportunistic infections and sexually transmitted infection services Referral for malaria and diarrhoea diagnosis and treatment	Increased uptake and “coverage” of services
	Adherence support	SMS (mobile phone text) and other adherence reminders Observation of ART ingestion and pill counting Tracing defaulters and acting as role models	Improved pick up rates of antiretroviral drugs and self-reported adherence rates to ART
	Needs assessment and disease screening	Nutrition screening and support TB screening (for chronic cough) Screening for opportunistic infections Monitoring domestic violence	Increased detection of TB cases among people with HIV.
	Livelihood training and support	Vocational training for vulnerable children: carpentry, metalwork, masonry and tailoring Income generation through microenterprises Gardening and farming	Improved social economic status of people living with HIV and vulnerable children
	Personal and palliative care	Home visits and home based care Food preparation and other household chores Dispensing drugs for opportunistic infections Wound care hygiene and dressing	Enhanced dignity and quality of life of people with HIV
Health systems-oriented themes	Service organization and delivery	Patient triage and accompaniment to clinics Translation, register filling maintenance HIV testing and condom distribution Delivery of ART and cotrimoxazole Laboratory sample processing	Reduced waiting times, improved patient flow and reduced workload of trained health providers
	Data collection, surveillance and reporting	Measurement and recording of vital signs Monitoring for ART side effects Monitoring HIV disease progression	Improvement in the filling of medical records

### Patient-related outcomes

#### Knowledge and literacy of HIV

Nine out of the twenty-one studies documented the role that CHWs were playing in providing HIV and general health education. CHWs educated families, primary caregivers and communities on symptoms and treatment of opportunistic infections in South Africa and Kenya; infection control, drug administration and reaction in Kenya and Uganda; and the importance of HIV testing before wife inheritance in Kenya [48–51]. CHWs also trained HIV-positive individuals on ART readiness and on the advantages and side-effects of ART in South Africa, Zambia and Mozambique [38, 51–55]. However, none of these studies examined the effect of these interventions in terms of knowledge of HIV among the populations of people living with HIV. Instead, one study focused on the effect of community-wide HIV education on stigma as perceived by people living with HIV involved in these studies. In this Kenyan study, the presence of CHWs was reported to contribute to a positive perception of people living with HIV in the community by demystifying HIV, interacting with people who had the disease and increasing their social visibility and acceptance [49].

#### Behaviour change

Although not consistently reported, CHWs activities were sometimes associated with behaviour change in relation to some pre-specified HIV-associated risky behaviours, as well as disclosure of HIV. The latter outcome is particularly important considering that people living with HIV involved in some of the studies were often not willing to disclose their HIV status, fearing stigmatization, discrimination and isolation, for instance in Kenya and South Africa [49, 56]. In this context, CHWs provided behaviour change counselling and facilitated HIV disclosure in South Africa [56, 57], resulting in better disclosure among patients with CHW support compared to patients who did not receive support (58% vs. 42%; *p*=0.005) [57]. Similar findings were observed in Ethiopia and Uganda in relation to improved disclosure [58]. In western Kenya CHWs counselled HIV widows regarding wife inheritance (a traditional custom associated with HIV transmission at the time of the study) [49], which motivated the widows to take up HIV testing before being inherited. An evaluation of these counselling sessions showed that widows either became “careful not to be inherited” or “preferred to have the [HIV] test done before being inherited” [49]. Based on concerns regarding the quality of counselling offered by CHWs, two studies investigated this outcome and found that the quality of counselling provided by CHWs in Malawi and South Africa was comparable or better than that provided by trained health care workers [38, 59].

CHWs performed a variety of behaviour change counselling, including pre- and post-test and ART adherence counselling in Kenya, South Africa, Malawi, Uganda, Lesotho and Zambia [38, 48, 52, 54, 57–63]; however, the scope of their involvement varied. CHWs in Kenya, Uganda, Lesotho and Zambia offered pre-test counselling, and then handed patients to trained health providers (typically nurses) to perform the HIV tests. In contrast, lay counsellors offered pre-test counselling and went on to perform HIV testing to 53,379 individuals at 14 different voluntary counselling and testing sites in Malawi, representing 41% of all HIV testing in the district in the two-year study period [59]. In South Africa, CHWs were a source of bereavement counselling for those grieving over HIV-related deaths during which they also discussed the future of potential AIDS orphans [48].

#### Uptake of HIV and other services

Several studies examined the effect that CHWs had on the uptake of HIV services, although this was often reported as “coverage” of services without objective measurements of coverage. CHWs in Malawi, and to a lesser extent in Mozambique, South Africa and Zambia, actively mobilized and referred patients to HIV services including voluntary counselling and testing, ART initiation, prevention of mother-to-child transmission (PMTCT) and treatment of opportunistic and sexually transmitted infections [48, 53, 59, 62, 64]; often leading to an increase in uptake of these services [53, 59, 65]. A few studies also reported an association between the deployment of CHWs and an increase in the uptake of other non-HIV interventions. For instance, studies from Malawi [59, 63], Mozambique [53], Rwanda [55] and Kenya [66] documented how CHWs screened for opportunistic and other co-infections, such as TB, diarrhoea, malaria and malnutrition among people living with HIV. In Malawi CHWs screened and referred 806 individuals for TB diagnostic services during their routine home visits to people living with HIV, of which 161 were diagnosed with smear-positive TB [59]. Some studies also reported that CHWs distributed condoms at the community level [51]. However, data regarding the uptake of services following referrals to other services (except TB) were sparse.

#### Adherence to ART

Adherence support to ART by CHWs, either during home visits or through mobile phone reminders, was a recurring theme across most studies. Most notable was the variety of strategies employed by CHWs to provide adherence support within community settings. In South Africa, Kenya, Uganda, Lesotho and Mozambique, CHWs supplied ARVs and other medications at the community level. They encouraged people living with HIV to take ART and observed ART ingestion during home visits in Kenya, Uganda, Rwanda, South Africa, Malawi and Zambia. They performed pill counting, used mobile phones in Uganda to remind patients to take their medication [48, 49, 51, 53–55, 57, 60–62, 64, 67, 68] and delivered up to one month's supply of ART to patients’ homes in Kenya and Uganda [51, 66, 68]. In Uganda and Ethiopia [58], peer counsellors acted as “expert patients” and facilitated adherence by acting as role models. In Uganda and Malawi, CHWs assessed and referred patients with ART-related toxicities, which were a frequent barrier to adherence [51, 63, 67–69]. In Mozambique, CHWs formed peer support groups of people living with HIV, who collected ART for those unable to attend their clinic consultations in person, and they also traced defaulters [53]. Adherence support for TB medications was frequently provided alongside that of ART [48, 50, 53, 55, 59]. Although a majority of studies documented the role of CHWs in adherence support, only one study examined the comparative effect that it had on adherence. In this South African study, patients with CHW adherence support were more consistent in picking up their medication, attaining a treatment pick-up rate of 95% compared to those without CHW adherence support (67%; *p*=0.021) [57]. Although treatment pick up may not necessarily translate to ingestion of the drugs, the study showed that the intervention arm was associated with better outcomes in terms of virological suppression (discussed below), suggesting that treatment pick up was a valid proxy indicator of adherence in this study.

#### Retention in care

Most studies found that the deployment of CHWs was associated with better retention of patients in HIV care. In Zambia an improvement in retention of ART patients from 85.4% to 100% was reported 12 months after the introduction of adherence support workers [52], although CHWs in this study were primarily adherence supporters and therefore the generalizability of this finding is uncertain. Nevertheless, a similar trend emerged in a randomized trial from Uganda that reported a two-fold difference in lost to follow-up rates at 24 months between patients who were supported by CHWs and those who were not (2.2% vs. 4.1% respectively) [61]. In Rwanda, a high rate of patient retention at 24 months (92.3%) was reported following the implementation of community-based adherence support [55], although there was no reported baseline for comparison in this cohort study. Three other studies [57, 60, 63] also reported improved retention. One notable exception from this trend was the study by Kipp *et al*. [68] which reported higher rates of loss to follow up in the intervention group (24.9%) compared to the control group (15.5%). However, this difference was not statistically significant.

#### Viral suppression

In circumvention of bias inherent in self-reported adherence, four studies looked at virological endpoints, with varying results. A cohort study in South Africa reported that adherence support from CHWs was associated with better viral suppression at six months [57], whereas two RCTs and one cohort study showed no difference in virological failure between intervention and control arms [51, 61, 67]. Although these studies used slightly different cut off points of 400 and 500 HIV-RNA copies/ml to determine virological failure, these findings suggest that virological suppression between trained health care workers and CHW groups was at least comparable.

#### Mortality

Similar trends were reported in terms of mortality. Two cohort studies showed that CHWs were associated with reduced risk of death [RR 0.22 (0.15–0.33)] in Malawi and improved survival after three years on ART (91.5% vs. 85.6%) in South Africa [54]. In contrast, an RCT in Uganda found no difference in mortality between home-based and facility-based models of HIV care at 26 months, even among very sick patients [67].

#### Social economic status and quality of life

Two qualitative studies [48, 49] showed that CHWs enhanced dignity and quality of life by creating a sense of belonging and companionship to people living with HIV in Kenya and South Africa, who would otherwise have felt isolated. A further study used Karnofsky score to compare functional impairment between intervention group and the control groups and found no difference at 12 months (*p*=0.46) [66]. Five studies described distinct roles in income generation that CHWs, some of whom were living with HIV, engaged in. For instance, in Mozambique groups of people living with HIV provided livelihood support to their peers through gardening activities [53]; in Kenya CHWs funded home-based care activities through proceeds from the sale of essential drug kits [49]; and in Malawi community volunteers trained 1694 AIDS orphans on vocational skills such as carpentry, metalwork, masonry, tailoring, bicycle repair, and cultivated vegetables and maize farms that provided food supplements for malnourished individuals [59, 63]. Elsewhere in Lesotho, CHWs distributed food packages [62]. Although these nutritional and income generating activities were well documented, it was less clear to what extent malnutrition and social economic status changed after these interventions.

#### Palliative care

Provision of personal care as a role of CHWs was less frequently described in the included studies. Notable exceptions were the two earliest studies in this review, in which palliative care was a central role of CHWs. The first study, from 2002, [48] noted the varied personal care that CHWs provided to people living with HIV during home visits, ranging from bathing, meal preparation, ambulation, bed baths, and mouth and wound care, to administering multivitamins and food supplements. The second study, in 2004 [49], found that CHWs in western Kenya assisted terminally ill clients with meal preparation, performed household chores, administered medication to treat opportunistic infections and performed needs assessment. Similar household activities were also performed by peer counsellors in Ethiopia and Uganda [58], although these activities were not central to their role as was the case in the earlier studies above.

### Health system outcomes

#### Service organization and delivery

Despite the heterogeneity of our data, there was similarity in CHWs' outcomes in relation to health service organization. In Zambia and Uganda CHWs were located at HIV clinics, where they guided patients through health facilities, reduced waiting times and improved patient flow [52, 60, 65]. Similarly, CHWs triaged and registered patients at clinics in Lesotho and Uganda respectively [60, 62]. In Lesotho and Kenya CHWs monitored and recorded patients’ vital signs during home visits [62, 66], which reduced the need for patients to visit health facilities significantly. Three studies examined the effect of shifting of tasks such as pre-test counselling from trained health providers to CHWs and found that it reduced the workload of trained health care workers in Zambia, Malawi and Uganda [52, 59, 60]. This was particularly pronounced in Malawi, where lay counsellors conducted 41% of all HIV testing performed in Thyolo district over a two-year study period [59]. Although CHWs processed laboratory specimens in Lesotho, the effect that this had on laboratory staff was not directly assessed [62].

In several studies CHWs acted as intermediaries between patients, health workers and health services; for instance, through language translation [62] or through social mobilization of communities to take up services [63]. They were “recognized for being close to the patients while acting as a bridge to the health system” [58] and for “improving patient-provider communication” [60]. One study documented CHW involvement in making decisions regarding the organization of health clinics in Lesotho [62].

#### Data collection, surveillance and reporting

There was an improvement in the filling of medical records when undertaken by CHWs in Zambia [65]. In this study, the error rate for CHWs filling medical records was 6.44/1,000 fields compared to 16.81/1,000 fields for trained health care workers. In another study from Kenya, CHWs utilized mobile technology tools to capture patient data [66], whereas elsewhere in Uganda, they filled in patient data in standardized forms [51]. These included data on adverse drug side effects, disease progression, vital signs [51, 55, 64, 66, 67] and, rarely, domestic violence [66].

#### Service cost

In relation to cost, the Ugandan home-based care trial [67] compared the costs between the community-based ART model involving CHWs and the usual facility-based care model and found that the intervention model was cheaper at USD 793 versus USD 838. However, the computation of cost-data was de-linked from the day-to-day activities of the CHWs, which raises an important limitation in relation to generalizability of these findings beyond the trial setting.

## Discussion

This systematic review of the role and outcomes of CHWs in HIV in sub-Saharan Africa found evidence from 21 studies that CHWs are performing a variety of roles, including counselling, HIV testing, home-based care, education, adherence support, livelihood support, screening, referral and surveillance activities. To a substantial extent, our findings are in line with reports from Asia and Central America showing that CHWs can increase the uptake of HIV services [70–72]. We found that CHWs also played an important role in supporting retention in care through defaulter tracing, adherence counselling, mobile reminders and collecting drugs from clinics; which improved retention rates in Zambia, Uganda and Rwanda. Patients who had been exposed to adherence support from CHWs had the same or less likelihood of virological failure in four studies, including two RCTs included in this review. To the extent of the data available in this review, findings appear to be consistent with the assertion that CHW outcomes are not inferior to those of trained health providers.

Evidence also suggests that CHWs can reduce waiting times, streamline patient flow and reduce the workload on health workers. In addition, CHWs can improve the human dignity and quality of life of people living with HIV by reducing HIV stigma, giving them a sense of belonging within their communities [48, 49, 56]. There is also some evidence that CHW-supported programmes may be more affordable than traditional health facility programmes [67], a benefit that has been reported in malaria and TB services [25, 26]. Another locus of economic benefits could emanate from CHWs involvement in income generating activities. Although this was not assessed in the studies included in this review, recent evidence from Uganda shows that engagement of CHWs in community-based ART programs can improve household incomes [73]. This is consistent with the findings from two recent reviews on task shifting [12, 31] showing that the deployment of lower cadres of health workers could lower costs and improve access to services without compromising patient outcomes. The use of mobile technology by CHWs in this review and more generally [74] could also contribute to better efficiency of community-based programs.

The results of this review support the World Health Organization's assertion that CHWs could be part of the solution to the human resource crisis in sub-Saharan Africa [27]. In keeping with the concept of task shifting, CHWs were found to ably perform tasks traditionally within the realm of qualified health workers, including HIV testing, filling medical records and processing laboratory specimens. One study suggests that CHWs were able to effectively perform some of these roles “because the community members are able to accept their teaching, and they become flexible and willing to be taught” [49]. The proximity of CHWs to their communities enabled them to demystify HIV and counteract social barriers such as stigma, while increasing uptake of HIV services [28, 56]. In these contexts, CHWs were seen as a bridge between the community and health facilities [38]. In Mozambique and South Africa, CHWs included people living with HIV who had had first-hand experience of negotiating through health services and, therefore, were able to provide appropriate support to their peers [53, 56], while being meaningfully involved in HIV care.

There is also some evidence that the quality of certain services provided by CHWs, such as counselling, may be as good as or better than that provided by trained health care workers in Malawi and South Africa [38, 59]. This is not to suggest that CHWs should always deliver these services in isolation or in every context. Rather, this suggests that CHWs should be part of multidisciplinary HIV teams, while performing the specific tasks they are best placed to. Differentiating which tasks CHWs can perform better than qualified health workers could inform task shifting policy. This could also have important implications in relation to skills training and capacity building of CHWs. Whereas by definition CHWs have limited or no formal training [75], this review found that some form of skills training is often needed to perform their roles effectively. In all except one study, CHWs were trained for short periods. For example, CHWs in Malawi provided effective HIV testing and counselling after two-to-three weeks’ basic training [59]. In addition several studies employed continued training and supervision of CHWs as part of quality assurance [51, 52, 61, 66, 67].

Despite their contribution to HIV services, a number of challenges facing CHWs were reported, some of which have been previously raised [76, 77]. These included a lack of nationally recognized training; poor recognition, remuneration and supervision; lack of psychosocial support; and poor involvement in decision making [38, 48, 49]. These challenges were often associated with perverse organizational cultures [14], economic hardships faced by developing countries [50], myopic strategic visions in health care and widespread lack of consensus on remuneration and recognition of CHWs [13, 78], often contributing to their demotivation and attrition [38, 76].

### Implications for HIV programmes

Implementers of HIV programmes should support further task shifting of HIV services by engaging CHWs to complement the overstretched health workforce, while at the same time implementing policies that mainstream CHWs into the wider health sector to mitigate their challenges. There is a growing body of evidence, including from this review, that task shifting to CHWs does not lead to worse outcomes for patients or health systems. Health authorities should therefore lead efforts to define and endorse CHWs’ roles, while providing strategies for training, supervision, remuneration, recognition, career progression and quality assurance.

Although some countries in sub-Saharan Africa, such as Malawi, Ethiopia and South Africa, have begun to integrate CHWs into their wider public health systems [28], the approach is highly variable. There is a need to formally recognise CHWs and clearly define their roles and responsibilities [76, 79], while ensuring that there is a coordinated approach to optimize their performance through quality management strategies [12, 28, 80]. Uebel *et al*. propose a compendium of elements that would be useful for national ART programs to consider in making decisions regarding CHWs including the model of ART delivery (facility versus community-based models), national regulatory framework for prescribing and dispensing ART, community-based supply of ART, and remuneration and evaluation of community-based service providers [81].

### Implications for future research

This review highlights several directions for future research. First, high-quality studies examining the potential for CHWs to deliver ART and other HIV services to different populations are required. These populations may include, children, men who have sex with men, sex workers, intravenous drug users, pregnant women and sero-discordant couples. In our review, one study assessed the role and outcomes of CHWs specifically among children. The rest of included studies focussed generally on all people living with HIV. Examining CHWs' roles and outcomes for specific population groups could inform appropriate CHW strategies for vulnerable populations. Second, more studies focussing on sustainable models and comparative costs of CHW interventions are needed. Finally, studies documenting successful approaches of mainstreaming CHWs into wider health systems are needed in order to share lessons learnt and inform such efforts in sub-Saharan Africa.

### Limitations of the review

This review has several limitations. Although multiple databases were searched, additional information may have been reported in conference abstracts and other grey literature sources that were not considered. Publication bias is inevitable for reviews describing models of HIV care in sub-Saharan Africa, because a considerable wealth of programme experience will remain undocumented and unpublished. In addition, although we intended to include studies with negative results, none were found; which may be due to publication bias. We may also have missed papers due to the use of methodological terms in our search strategies, although an inclusive approach to capture qualitative and all types of quantitative studies strengthened our study. Furthermore, while both English and French language publications were searched, all studies included in this review were conducted in east and southern Africa. No papers from West Africa were found. Finally, some of the included studies had quality limitations, such as insufficient attention to minimizing bias, or controlling for confounding. Despite these limitations related to quality, and in-order to ensure all relevant studies were included, no studies were excluded from our review based on quality.

## Conclusion

To sum up, our review found that CHWs perform a variety of roles in HIV prevention, treatment and care; with no evidence that patient outcomes and quality of care are compromised. CHWs may also have positive impacts on HIV service organization, delivery and cost. However, to be scalable and sustainable, CHWs need to be better integrated into wider health systems to ensure their contribution is formally recognized and remunerated.
